# A Systematic Literature Review of Handheld Augmented Reality Solutions for People with Disabilities

**DOI:** 10.3390/s22207719

**Published:** 2022-10-11

**Authors:** Matea Žilak, Željka Car, Ivana Čuljak

**Affiliations:** Faculty of Electrical Engineering and Computing, University of Zagreb, 10000 Zagreb, Croatia

**Keywords:** augmented reality, human-computer interaction, accessibility, people with disabilities, handheld devices, digital inclusion

## Abstract

Mobile applications on smartphones and tablets have become part of our everyday lives. The number of augmented reality (AR) technology applications is also increasing. Augmented reality has proven to be effective in various areas of human life, from education, marketing, and training to navigation. All people have the right to access information and use available technologies, but not everyone has the same opportunities. To contribute to the digital inclusion of people who are often disadvantaged in this regard, we should think about the accessibility of digital technologies, including mobile augmented reality (MAR). The specificity of MAR is a new way of human–computer interaction compared to traditional mobile solutions. The objective of this review paper is to analyze the handheld AR solutions developed for people with different disabilities to identify accessibility challenges related to interaction when performing different tasks in AR. It also explores and presents accessibility features and other accessibility best practices, as well as potential future research directions related to the personalization and customization of such solutions for individuals. The results of this literature review can contribute to the creation of accessibility guidelines in the field of handheld AR and encourage the development of accessible AR solutions that can benefit not only people with disabilities but also people without disabilities.

## 1. Introduction

Today, technological progress is taking place at an exponential rate, allowing technologies to move more rapidly from research laboratories to the marketplace. New ideas and products are accepted by new customers faster than before. Computers, smartphones, the internet, and social media are examples of the technologies in use today with rapidly increasing adoption rates. The exponential progress of enabling technologies in terms of computing power, big data, device connectivity, and internet performance, has enabled the development of solutions based on emerging technologies that are robust enough to be valuable to consumers and useful to the public. To develop a mainstream solution that fulfils its role, the user-centered approach needs to be followed; otherwise, there is a risk of having to re-engineer a solution because it does not reflect the specific needs of a certain group of people, e.g., people with disabilities [1]. When talking about the integration of mainstream and assistive technologies, it is worth mentioning that the implementation of accessibility features in everyday applications has proven to be beneficial for people without disabilities as well. For example, subtitles in a video are useful not only for people with hearing impairments but also for people experiencing an ear infection (temporary disability) or people in a noisy place (situational disability) [2]. Accessibility, in general, can be achieved if principles of universal design are followed, which is defined as a “design of products, environments, programmes and services to be usable by all people, to the greatest extent possible, without the need for adaptation or specialized design” [3]. Considering the different needs and abilities of people when designing a product, equal opportunities for its use can be achieved, as well as the inclusion of people in different aspects of life.

According to studies described in [1], the majority of people with disabilities have an open and optimistic attitude towards new and emerging technologies. Virtual reality (VR) and augmented reality (AR) are technologies that enable new forms of human–computer interaction and whose industries are growing rapidly. The main difference between these technologies is that VR immerses the user completely in a synthetic environment, i.e., the user cannot see the real world around him, while AR supplements reality and allows the user to experience the real world with virtual objects superimposed on or composed with the real world [4]. According to [5], AR can be further refined based on the level of local presence, ranging from assisted reality to mixed reality (MR). While in assisted reality content is perceived as clearly artificial and overlaid (low local presence), in MR users experience virtual content as actually present in their physical environment with the possibility to interact with each other (high local presence) [5].

VR is most known for its major use in gaming as it allows gamers to have a full-immersive experience in the gaming world. Having in mind ubiquitous computing and that people are naturally more directed towards the real world instead of virtual, AR excels as a more appropriate technology since it provides a simple and immediate user interface to an electronically enhanced physical world [6]. The most commonly accepted definition of AR, described in [4], says that a system is considered AR if it contains the following three characteristics: combines real and virtual, interactive in real-time, and registered in 3D (i.e., correct alignment of the virtual world with the real one) [4]. This definition does not limit AR to specific technology such as a head-mounted displays (HMDs) but also allows monitor-based interfaces, see-through HMDs, and various other combinations of technologies [4]. Virtual information that could be overlaid on top of the user’s real-world view can be textual, symbolic, and graphical (2D and 3D graphics) [7]. Depending on the method of augmentation that AR displays employ, three categories of AR displays can be distinguished: optical see-through displays, video see-through displays, and spatial projection [6]. Monocular video see-through displays include smartphone- and tablet-based handheld AR, and together with different optical see-through displays (mobile HMDs) are known as devices that belong to mobile AR (MAR) [6,8]. Many head-worn displays are still considered cumbersome and high-cost, so handheld displays are (currently) more socially accepted [8] although they bring potential issues such as arm strain after long use.

Despite ongoing challenges, AR holds the potential of being used in many different fields. Apart from applications for specific domains, such as industry and construction, maintenance and training, or medicine, AR can be used by a broader audience in use cases that involve everyday tasks, such as finding information about places of interest in their surroundings, navigation and support while following a route, augmentations in advertising and commerce, helping consumers make buying decisions, and support while learning, as well as AR gaming, which is expected to be the consumer use case with the most investment in 2024, together with VR gaming and VR video/feature viewing [9]. Most of these applications have become available thanks to advancements in mobile computing and the proliferation of smartphones. Although the first handheld AR system (running on a personal digital assistant) dates back to 2003 [10], it was not until 2016, when the AR mobile game Pokémon GO [11] was released, that handheld AR became popular and familiar to the masses.

Considering the benefits that AR can bring as a new way of human–computer interaction in the real world and the fact that AR applications are becoming ubiquitous and part of our everyday life, the question that imposes itself is: “Will everyone be able to experience engagements with augmented reality?”. To take full advantage of the benefits that advancements in the AR domain can bring to the world, one needs to think about the accessibility of different products and services that are being developed, i.e., whether all users be able to use them regardless of their capabilities and limitations. In addition, to achieve full accessibility, compatibility with assistive technologies needs to be assured. For example, a screen reader is an assistive technology whose compatibility with devices, such as smartphones and computers, is imperative for visually impaired persons to perceive information that appears on the websites or different applications running on the devices. Since there are over one billion people worldwide living with some form of disability [12], the accessibility of new products, especially those that are predicted to be widespread and part of everyday life, imposes itself as a must-have feature to improve the social inclusion of people with disabilities. This paper presents one step towards an inclusive society by investigating the current status of accessibility in the field of handheld AR solutions for people with disabilities.

In this paper, a systematic literature review (SLR) of existing handheld AR solutions for people with disabilities is performed. This SLR allows us to obtain an overview of AR solutions developed for people with disabilities in terms of interaction tasks and techniques required by users with different disabilities, to identify accessibility challenges that need to be addressed in future work. Thus, the focus is not on describing the functionalities of solutions, but rather on exploring different interaction techniques required to perform AR tasks, with an emphasis on multimodality and alternative ways of interacting with virtual objects. This is very important because only if appropriate input and output modalities are found for each person can the full potential of AR-based solutions be realized. Although AR is mostly associated with visual augmentations, other sensory modalities can also play an important role. For example, tactile technologies can now enable interfaces that can be invaluable for people with different disabilities. For people with motor difficulties participating in home-based telerehabilitation, providing tactile feedback can be useful to improve motor control performance and learning [13], while for people with visual impairments, interactive tactile maps, diagrams, and real-world objects augmented with audio feedback can be very beneficial in educational settings [14].

The focus in this review is put on handheld displays, i.e., on the techniques developed for touch screens that involve 2D gestures and (multi)touch, but other input modalities such as voice command and device-based interaction are also considered. Although AR can augment the real world not only visually, but also with sound, touch (haptics), smell, and taste (multimodal augmentation), in this paper the focus is on handheld displays integrating mostly visual modality (visual augmentations). One can think that in this way people with visual impairments are excluded, but visual AR can be made accessible to them as well if the support for assistive technologies is implemented. With respect to the goal of this paper, the following research questions (RQ) are defined:

1. RQ1: What types of handheld AR solutions are being developed for people with disabilities?
2. RQ2: What common interaction tasks are required in handheld AR solutions for people with disabilities?
3. RQ3: What interaction techniques are being used for performing common tasks in handheld AR solutions for people with disabilities?
4. RQ4: What accessibility features have been implemented in handheld AR solutions for people with disabilities?
5. RQ5: What research methods were applied in the design of handheld AR solutions for people with disabilities?

This paper is organized as follows. Section 2 introduces the topic of accessibility and the current status of accessibility guidelines for web, mobile, and AR solutions. In Section 3 the process of performing SLR is described. Section 4 then presents the literature review results. Discussion of the results and future research work are presented in Section 5. Finally, the conclusion is presented in Section 6.

## 2. Background

Accessibility is defined as a measure that indicates how much some product, service, or environment is suitable for all users, including people with disabilities as well as elderly people. Since several types of disabilities can be distinguished (auditory, cognitive, neurological, physical, speech, visual) where one person may have more than one disability, it is not always easy to meet all the needs of people with disabilities. Although they are a heterogeneous group of users and their requirements are often contradictory, we need to strive towards the fulfilment of as many requirements as possible. The key approach to achieving accessibility is User-Centered Design (UCD) which is based on the active involvement of users to improve the understanding of user and task requirements, and the iteration of design and evaluation [15]. Besides accessibility, usability and user experience (UX) are very important factors in the process of accepting the product by the masses. All three terms are closely related to the UCD approach. Although many use the terms usability and UX interchangeably, the term UX is much broader. The user experience is defined as “user’s perceptions and responses that result from the use and/or anticipated use of a system, product or service” [16], while the usability is defined as the “extent to which a system, product or service can be used by specified users to achieve specified goals with effectiveness, efficiency and satisfaction in a specified context of use” [16]. An essential part of the UX design is the interaction design which aims to design an interactive product that allows users to achieve the goal in the best possible way, i.e., the focus is put on the way users will interact with the product depending on the users’ needs, limitations, and context [17]. Since AR technology requires new ways of interacting with a user, interaction design plays an important role in achieving quality and accessible interactive AR solutions.

The importance of digital accessibility is recognized worldwide as there are many legislation and public policies that obligate public sector bodies to make their websites and mobile applications accessible [18,19]. There are also different accessibility standards and guidelines available to make the web accessible to people with disabilities, such as Web Content Accessibility Guidelines 2.0 (WCAG 2.0) intended for web content, authoring tools, and accessibility-evaluation tool developers [20]. These guidelines can also be applied to a mobile environment, i.e., to achieve accessibility of websites and applications when using mobile devices. For mobile accessibility, it is important to consider mobile-related issues such as small screen size, touch-target size and spacing, touchscreen gestures, grouping operable elements, easy methods for data entry, and changing screen orientation [21]. These guidelines can also be applied to augmented reality applications for mobile devices such as smartphones and tablet devices; however, AR-related characteristics (e.g., user movement in a physical environment, environmental limitations, interaction while holding the device) need to be considered and investigated to fully understand the specifics related to the accessibility of AR-based applications for mobile devices.

Important guidance can be found in the domain of extended reality (XR). XR is an umbrella term for VR, AR, MR, and all future immersive technologies that can merge the physical and virtual worlds [22]. XR Accessibility User Requirements (XAUR) [23] is a working draft document that aims to outline the diversity of current user needs related to accessibility in XR and the potential requirements for fulfilling those needs. One of the accessibility challenges highlighted in the XAUR document is related to providing accessible alternatives for content and interaction, which means that customization of the content or aspects of a user interface from one mode to another is provided so that various user needs are supported [23]. In addition to understanding which input or output modality best suits a user, it is important to distinguish all other less obvious factors that affect the usability and user experience of an XR-based solution. Regarding handheld AR solutions, some of the factors may be, for example, audio clarity, text size and contrast, button-layout options, movement requirements, interaction with the environment, or blending virtual objects with the real world. Interaction techniques implemented for head-worn displays are not suitable for handheld devices, since at least one of the user’s hands is needed to hold the device. Although the touch-screen interface is one of the widely used interaction methods in handheld AR, this method can often lead to unprecise interaction or unexpected errors due to inaccurate coordination between both hands [24]. Thus, additional techniques are proposed by the researchers, one of which is the freeze interaction technique which allows the user to freeze the AR view or virtual object and interact with it [24]. These and other factors collectively affect whether an AR application will be an accessible and enjoyable experience for users regardless of their abilities. Once the necessary accessibility features are recognized, it is important to understand how to categorize them and where to place them (e.g., in a menu) so that they are discoverable in multiple contexts [23].

Since AR is a rapidly evolving field and many users often find themselves experiencing AR for the first time, it is important to develop products that are intuitive and include well-explained instructions or illustrations. There are examples of demonstration applications and design guidelines for developers that cover different AR principles and patterns, such as [25,26,27], but these are more design guidelines that relate to the environment, virtual content, real-world movement, and the user interface in general. The aspect of designing an accessible AR application from the beginning is missing, which may lead to touching up later or developing an alternative version of the application, which is not in line with the best practice of considering accessibility features early in the development. A guideline related to a particular disability, mentioned in [25], points out that an alternative way to use the application is needed if a user is not able to move around, e.g., let the user move and rotate an object. This brings us to think that more specific and detailed guidelines for making AR applications accessible to users with different needs are required for developers and designers of AR solutions.

To be able to offer such guidelines that will consider all the factors affecting accessibility, extensive research needs to be performed in this field. Although the emergence of AR impacted the growth of research in AR in general as well as in the accessibility domain, the interest in AR for people with disabilities remained relatively low. According to [28], research projects in this domain mostly focus on accessible AR interface design with two aspects: one tends to focus on the best way to gather real-time spatial data into an accessible representation, and the other one tends to be the delivery of that information via different modalities, while a significant research area in the literature is focused on how AR can be used as an instructional tool for people with disabilities completing real-world tasks. Besides having AR-related research that looks at specific applications for people with different range of abilities (e.g., for students with paralysis and cerebral palsy to motivate them towards physical activity [29]), it would be worthwhile to direct research towards having accessible, mainstream AR solutions that could be used by people no matter the abilities. According to [30], researchers on accessible computing have already taken the effort to personalize interaction mainly for traditional audio-visual media (not immersive media). Existing research investigated interface personalization based on user profiles and for users with different ranges of abilities; however, they do not have any objective mapping between users’ abilities and interface parameters, especially in XR systems [30]. An example of a community committed to making XR accessible to people with disabilities is XR Access [31]. Although they investigated accessibility issues of immersive media and identified different areas to improve and promote its accessibility, specifics related to AR accessibility are not discussed in detail [30,32].

To contribute to the essence of the AR accessibility problem, it is necessary to fully understand the needs of users with disabilities regarding interaction techniques in AR and the appearance of virtual objects in the real environment as this is the main difference between AR applications and usual mobile applications that already have established interaction mechanisms. This systematic literature review (SRL) contributes to a better understanding of the interaction-design principles for people with disabilities in handheld AR solutions by providing an analysis of research studies and papers describing AR solutions for people with disabilities, focusing on the different interaction techniques required in AR. The results of this SRL may also contribute to the creation of accessibility guidelines in the field of handheld AR and encourage the development of accessible AR solutions. Further research and development in this area motivated by this paper will also have an impact on expanding the field of digital inclusion. The following sections describe the systematic process of the literature review and the results relevant to the field of accessibility of AR for handheld devices.

## 3. Systematic Literature Review Process

A systematic literature review (SLR) in the field of accessibility for handheld AR solutions is conducted following the PRISMA method [33]. The overall goal of this review paper is to get one step closer to accessible AR for all and identify features impacting AR accessibility on mobile devices the most. Therefore, research questions have been defined accordingly and the SLR process is planned. The SLR process includes four phases which are described in this section in detail.

The first review phase is the *Identification* phase, whose goal is to identify the number of papers according to the defined search strategy. Our search strategy includes searching the *Web of Science Core Collection* database, which is a trusted, high-quality collection of journals, books, and conference proceedings available on the *Web of Science* multidisciplinary platform with more than 74.8 million records [34]. The search is performed with the following search query A AND (B OR C), where:

- A equals to (“augmented reality” OR “AR”) in the title (TI);
- B equals to (“augmented reality” OR “AR”) AND (“mobile” OR “handheld”) AND (accessibility OR disability OR impairment OR special needs) in the abstract (AB);
- C equals to (“augmented reality” OR “AR”) AND (accessibility OR disability OR impairment OR special needs) in the author’s keywords (AK).

**Figure 1:**
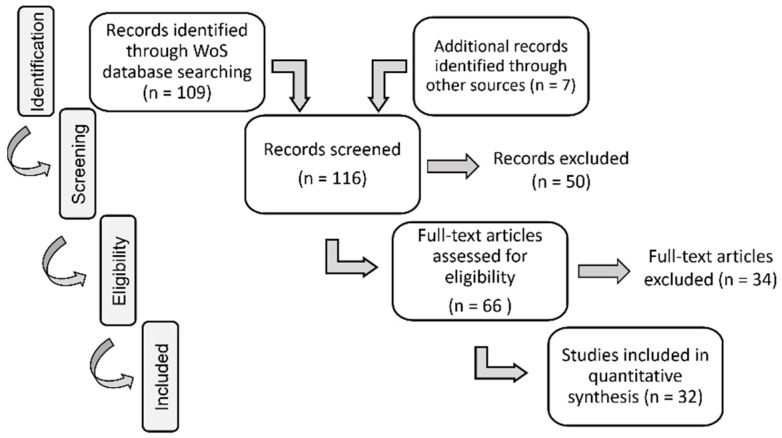
The PRISMA flow diagram with indicated number of publications in different phases of the systematic literature review.

The database search results were concluded in January 2022, considering publications written in English as well as publications published between 2010 and 2022. Additionally, document types included in the performed search are proceedings papers, articles, and early access papers. The initial database search retrieved 109 publications. An additional seven records relevant to the topic were identified through other sources (e.g., manual search through references of relevant publications), and thus were included from the beginning of the process. Figure 1 shows the flow diagram with the number of records in different phases of the systematic review.

The second phase of the systematic review is the *Screening* phase in which the publications were reviewed by title and abstract. The main inclusion criterion in this phase was: the publication presents a solution that is based on handheld AR and is related to people with disabilities in terms of inclusion (social, educational, or other), development for specific disability (assistive tool), or universal access in general (e.g., solutions for mainstream usage). Publications were excluded in accordance with the following exclusion criteria: (i) accessibility is mentioned in the context not related to people with disabilities, i.e., the term is used in the context of affordability by price or access to information; (ii) the solution described in the publication is not related to handheld AR but rather to spatial AR or head worn AR, i.e., smart glasses or HMD, or it uses additional sensors like Kinect for recognizing hand gestures; (iii) AR solution is considered from the perspective that is not related to people with disabilities, e.g., in connection with network latency, machine learning, cloud computing, cultural heritage, medical education; (iv) the solution described is VR- or MR-based, not AR (mostly virtual worlds or solutions used with wearable devices); (v) there is no concrete solution mentioned or any AR details; (vi) AR is not the main technology used or the main functionality of the solution/system, e.g., the publication is more related to robotics or BCI (brain–computer interface) research. If reviewing the title and the abstract did not give enough information to decide whether the publication is relevant for the review, that publication was included in the full-text eligibility assessment. In this phase, 50 publications from the search track were excluded.

The third phase of the process for selecting publications to be included in a systematic review is the *Eligibility* phase. A total number of 66 publications were included in the full-text assessment for eligibility. The exclusion criteria in this phase were the following: (i) the solution presented in the publication is not based on handheld AR (when this was not obvious from the title and abstract); (ii) there is no description of an AR application or details related to interaction technique(s) used, e.g., the publication only provides examples of situations/use cases in which the AR application can be used; (iii) the description of an AR solution provides guidelines on how it can serve as an authoring tool for educational content, and not how it can be used by people/students with disabilities; (iv) the AR application is described in its early stage of development or planning. After full-text reading, some publications were identified as connected research or continuations of a study, where, e.g., the older publication is a conference paper, and the other one is a journal article on the same topic. Thus, in that case, only journal article was considered and included in the analysis.

In the last phase, we concluded the number of 32 *Included* publications for the SLR that include seven additional publications included from the beginning of the process that remained eligible to the end of this phase. An overview of publications selected for the SLR, as well as the results of their analysis, is provided in the next section.

## 4. Systematic Literature Review Results

**Figure 2:**
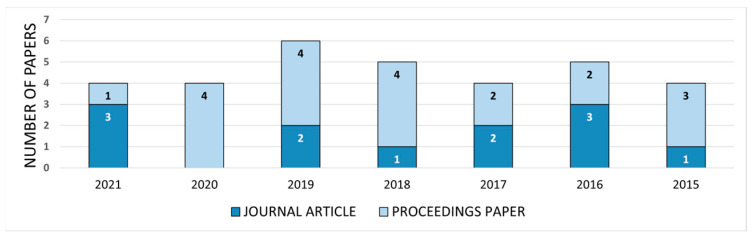
Distribution of publications by published year and type of publication.

At the beginning of the analysis, publications were categorized by the year of publication and publication type. Although the literature search performed considered publications from 2010 onwards, among the papers included in the analysis there were no papers from the period from 2010 to 2014. Since 2015, the number of papers relevant to the topic of AR accessibility is between 4 and 6 through the years, with a slightly higher number of conference papers (62.5%) compared to journal articles (37.5%). Given this fact, we can say that the importance of this research topic has attracted the attention of the research community since 2015, which is not surprising because only then did handheld AR become more popular and affordable among the general public. The distribution of papers by year and type of publication can be seen in Figure 2.

In the next sections, we bring results based on the literature review of publications included which provide answers to the research questions defined. Each publication is read thoroughly, and data were extracted into a Microsoft Excel spreadsheet for analysis, covering aspects of the following areas: (1) AR characteristics used in solutions developed for people with disabilities (including tracking technology, triggers, and augmentations); (2) Common interaction tasks required in AR handheld solutions for people with disabilities; (3) Interaction techniques used for performing common tasks in AR solutions for people with disabilities; (4) Accessibility features identified in AR solutions developed for people with disabilities (including alternative options for interactions and content as well as design guidelines); (5) Research methods applied in the design and evaluation of handheld AR solutions for people with disabilities.

### 4.1. RQ1: What Types of Handheld AR Solutions Are Being Developed for People with Disabilities?

In this section, we classify AR solutions described in publications by characteristics specific to AR mobile tracking systems. Tracking refers to the registration (alignment) of a virtual object in the real world and continuous tracking (measurement) of the viewer’s position and orientation in relation to some “anchor” in the real world [35]. Depending on the technology used, there are several common tracking techniques used in mobile AR solutions. Vision-based tracking is one of the most popular categories of tracking techniques since mobile devices such as smartphones and tablets fulfill minimal hardware requirements necessary for this tracking approach. Computer vision techniques used in this category can provide marker-based and markerless tracking at real-time frame rates [35]. Artificial landmarks that are added to the environment to aid in registration and tracking are called fiducials and represent the tracking target (i.e., trigger) for the marker-based tracking type [35]. Table 1 shows the categorization of AR tracking technology characteristics that can be determined for every AR solution analyzed. The first column in the table represents the AR tracking technology category, while the second column represents a more specific tracking technology type based on the way the virtual content is registered and tracked in the real world (by the sensor/technique used) [35,36]. Examples of tracking targets for each tracking technology are shown in the third column of the table. The markerless tracking type includes natural feature tracking which detects features in the captured images which are unique in their surroundings, e.g., points, corners, and the intersections of lines [35]. One of the techniques known in the markerless category is based on the SLAM (Simultaneous Localization And Mapping) process. SLAM enables the device to understand its relative position in the world and to be aware of its surroundings, such as the floor, walls, or other barriers. It constructs a real-time 3D map of an unknown environment that remains consistent during movement [37]. The GPS tracking technique is also very popular since it enables positional tracking in outdoor environments. With accurate location registration of users’ points of interest, valuable information can be provided to users. Being equipped with various inertial sensors, such as accelerometers and gyroscopes, as well as cameras and GPS, mobile devices present new opportunities for hybrid tracking that could benefit AR tracking [35].

The analysis of the publications by the tracking technology type used in the AR solution shows that the largest number of solutions is based on vision-based technology (about 78% of solutions). Three solutions are based on location-based tracking technology (about 9.5%) and the rest of the solutions fall into the category of hybrid solutions (12.5%). Out of vision-based solutions, only five of them (20%) use the markerless type of technology, i.e., they use the environment or real-life objects as tracking targets, while the rest of them (80%) use marker-based technology. Most of the triggers for the marker-based solutions are visual markers or images related to study material (e.g., in a textbook/electronic book, organized in booklets, on flashcards, or on learning objects), while other visual markers are based on the QR code format, with images of objects prepared in advance so that they can be recognized, and one solution with AR markers on a tangible object (AR cube). All the location-based solutions use a GPS signal as a primary tracking technology and all the applications serve as an assistive tool for wayfinding and improving navigation skills. Regarding hybrid tracking technologies used in a certain solution, most of them are related to tracking the GPS signal in combination with inertial tracking, e.g., tracking the device’s orientation and direction of pointing, but also a combination of GPS signal with pattern recognition is used. There are no solutions that are based solely on inertial tracking technologies because inertial tracking targets are typically used in combination with other primarily used tracking techniques to improve recognition and tracking accuracy.

**Figure 3:**
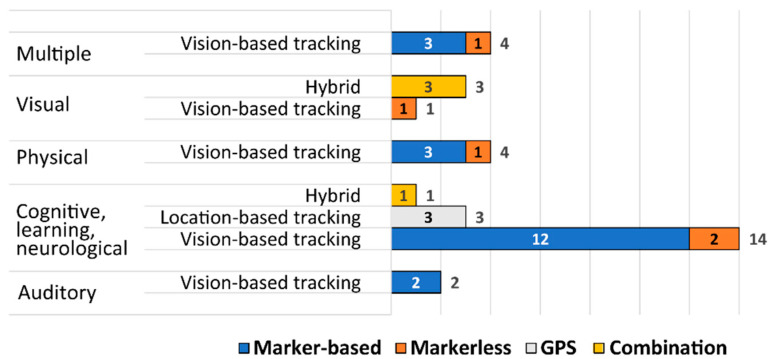
Distribution of AR handheld solutions by the disability type and type of tracking technology used in the solution.

The distribution of AR handheld solutions based on a certain tracking technology type categorized by disability types for which the solution is developed can be seen on the graph shown in Figure 3. Most solutions have been found for the category of *Cognitive, learning, and neurological disabilities* (CLN), i.e., 18 solutions or about 56%, which is understandable because this category includes the most diverse group of users compared to other types of disabilities. Solutions for people with attention deficit hyperactivity disorder (ADHD), autism spectrum disorder (ASD), intellectual disabilities (i.e., Down Syndrome), mental health disabilities, perceptual disabilities (i.e., dyslexia), and cognitive impairments (e.g., elderly people) are included in this category. The *Multiple disabilities* category refers to solutions that are not strictly defined for a particular disability but can be used in different cases and are thus designed, e.g., solutions are intended for children with special needs who require a different type of support, or the elderly with various impairments (motoric or visual). There are four such solutions, and the same number of solutions, or 12.5%, have been found for the categories of *Physical* and *Visual disabilities*. The least number of solutions (only two) have been found in the category of *Auditory disabilities*. In almost all categories of disabilities (besides the *Visual disabilities* category), solutions based on visual tracking technology prevail, especially marker-based solutions. For auditory disabilities, marker-based solutions are the only ones found, while for physical and multiple disabilities there is one markerless solution in addition to marker-based solutions. In the CLN category three solutions based on GPS signal can be found, two markerless solutions and one hybrid solution. In the category of visual disabilities, there are three hybrid solutions and one markerless solution, i.e., no marker-based solutions, which is also understandable because, to augment the real world, you need to point a device towards a marker or other trigger which is difficult for the blind and low-visioned people.

**Figure 4:**
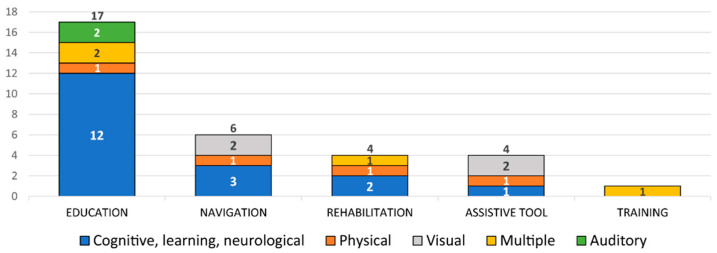
Distribution of AR solutions by disability and application-domain categories.

Given the application domain for which a solution was developed, most of the solutions belong to the educational application category (about 53%), with most being for the CLN category, while solutions for the visual impairment category were not found. The next application category by a number of solutions represented (about 19%) is navigation (or wayfinding assistance), for which no solutions for auditory disabilities were found. The same number of solutions (12.5%) can be found for the application areas of rehabilitation and assistive tools. Again, there are no solutions for auditory disabilities, and there are no solutions for visual impairments in the rehabilitation area. Only one solution (3%) belongs to the category of training application domain category for multiple disabilities, i.e., for elderly people who may have different cognitive and physical abilities. The graph representing a described distribution of AR solutions by type of disability, categorized by area of application, can be seen in Figure 4.

**Figure 5:**
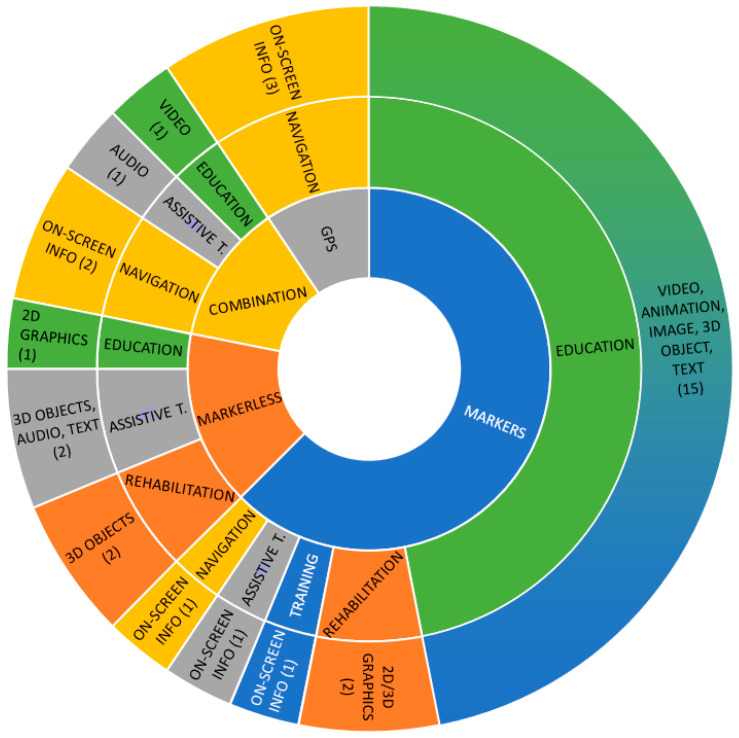
Distribution of AR solutions based on the primary AR virtual content used (the farthest circular wreath on the graph) and categorized by AR tracking technology type and application domain.

Most solutions use visual output as the primary modality with accompanying audio or voice reproductions. Types of virtual visual content used in AR solutions are various, from 3D objects, images, animations, shapes, videos, and text, to on-screen information, and most are independent of the tracking technology type, except for GPS-based solutions in the navigation domain, where on-screen information, such as different 2D graphics, text, or symbols indicating the right path and information about location and distance are used as augmentations. In educational applications, different combinations of AR content are used regardless of the tracking technology type. Furthermore, 2D and 3D graphics are mostly used in rehabilitation applications, while assistive tool augmentations depend on the disability category. For example, assistive tools for visually impaired and blind people combine 3D objects and audio, where the audio output is the primary modality, while for dyslexic people the augmentation is an alternative display of text and background. The only solution in the training domain uses additional information to instruct workers during operation. Figure 5 shows the distribution of the augmentations used in the AR solutions by type of tracking technology and application area.

### 4.2. RQ2: What Common Interaction Tasks Are Required in Handheld AR Solutions for People with Disabilities?

Regarding the way the user interacts with the virtual content in an AR application, we can distinguish the following AR interface types: *Information Browser*—an interface for showing AR information in the real world; *3D User Interface (UI)*—uses 3D interaction techniques to manipulate content in space (through controllers); *Tangible UI*—uses real objects to interact with AR virtual content; *Natural UI*—uses natural body input such as freehand gestures; *Multimodal UI*—uses combined speech and gesture input [35]. Almost all AR solutions analyzed are AR information browsers (96.9%), i.e., only one solution is a tangible UI [38]. Solutions with an information-browser-type interface include displaying AR content anchored on the real world and the appearance of virtual content on the screen after activation (e.g., after scanning a marker, instruction text pops up on the screen).

When talking about accessible alternatives for interactions in handheld AR, it is important to distinguish the types of interactions required by the users for performing constituent tasks in mobile AR applications. By using an existing taxonomy of constituent tasks (from [39]), different types of interaction tasks appearing in AR solutions for people with disabilities are identified. Table 2 shows identified tasks per AR solution categorized by disability type. Since tasks belonging to the *Observing Virtual Content* category are identified in every AR solution (whether it is perceiving information about a virtual object visually or auditory), they are not considered in the table. In general, all AR solutions include some type of task from *Establishing Physical/Virtual Correspondence* category since this is necessary to create a relationship between the real-world and virtual content appearing on the screen. The biggest percentage of solutions from this category (62.5%) belongs to the *Scan marker* task type which is in correspondence with the fact that most of the AR solutions analyzed are based on markers. In this task category, the same percentage of solutions (15.6%) include *Scan location* and *Scan environment* tasks, while 12.5% of solutions include *Scan object* tasks. None of the solutions from CLN and auditory disabilities categories use scanning of the environment as one of the constituent tasks. Additionally, *Scan location* is used only in solutions from categories of CLN and visual disabilities while *Scan object* is used only in solutions for CLN and physical disabilities. The second most popular task type is the *Trigger effect* from the *Activating Virtual Content* category of tasks; 21.9% of solutions include this task. This task type includes actions such as triggering sound effects, animations, and additional content (text or sound information). In this task category, there is also the *Select* type of task which is included in 15.6% of solutions, most of them in the visual disabilities category and related to the selection of a certain location. From the *Transforming Virtual Content* category, the most popular task types are *Move* and *Rotate*, since 12.5% of solutions include this task type while 9.4% of solutions include the *Scale* task and only one solution includes the *Color* transformation task. The *Place object* type of task from the *Placing Virtual Content* task category is included in 12.5% of solutions, and it is not specifically related to any of the disability categories.

### 4.3. RQ3: What Interaction Techniques Are Being Used for Performing Common Tasks in Handheld AR Solutions for People with Disabilities?

In this section, we bring interaction techniques identified for AR interaction tasks together with the description of the input method (procedure) required to perform the task. As mentioned before, the emphasis in this paper is put on handheld AR, which means that the device on which the AR solution is being used is either a smartphone or tablet. An AR solution that uses a laptop with a webcam is also considered if the same interactions can be applied to handheld devices. Most of the solutions considered in this review are used on handheld devices, so it is understood that a user needs to hold the device with one or both hands. This is emphasized later in the description of the input method if the procedure, besides holding a device, includes a precise pointing from a user to, e.g., scan a marker, image, or specific location, or if the environment around the user needs to be recognized or established to continue using the application. Two solutions are exceptions where the user does not interact with the device but brings the markers or tangibles in front of the device camera, while the device is positioned on the stand, or a device is a laptop with a webcam. Most of the interaction techniques are common for mainstream AR solutions, except for specially designed interactions for visually impaired users, i.e., screen-reader users, as is the case in [39]. The researchers in [39] developed prototype applications with accessible alternatives to the current AR interactions and conducted a user study with ten participants (blind and low vision). The results showed that AR experience is possible for blind people.

Interaction techniques identified in AR solutions are summarized in Table 3 where they are grouped by AR interaction task type and disability category. It should be noticed that solutions from different AR task categories include different interaction techniques since the aim of the tasks is different. The tasks from the *Establishing Physical/Virtual Correspondence* category have a similar goal and thus interaction techniques for accomplishing them are similar (*Point(ing) to…*), but again their input methods differ depending on the application domain, as can be seen from the table. In some solutions, it is enough to find a surface where an object will be automatically placed ([69]), while setting the scenario elsewhere is necessary to begin with the application ([64]). The category that has the most diverse possibilities of interaction techniques is *Transforming Virtual Content* as manipulation with virtual content can be performed in various ways. Most of the solutions from this category include the manipulation of virtual objects with gestures on the screen, such as drag and drop, slide gesture (swipe), tap, or pinch to zoom, as well as with UI interactions, such as a slider or button.

Manipulating virtual objects can be a very complex task (considering that at the same time a user needs to hold a device), so the way of performing an interaction can play a big role in the accessibility of a solution. Only a few solutions provide alternative interaction techniques or input modalities for virtual object manipulation. One solution provides different types of UI elements for performing the same actions [66], while two solutions provide voice commands in addition to the usual screen input gestures [60,62]. In the AR task categories *Placing* and *Activating Virtual Content*, different interaction techniques for screen reader users are identified depending on the application domain (retail or education) [39].

Although the input method for the interaction techniques *Click* and *Tap* is similar, the difference between them is the target on the screen with which a user interacts. When a target is static on the screen, such as the UI button or other virtual content that appears after activation, the user clicks on it, while for targets that are anchored in the real world, the user must hold the device in a certain direction to have the target in the camera view and then tap on the screen where the target is visible. These interaction techniques occur in both AR task categories: *Transforming* and *Activating Virtual Content*.

Other interaction techniques mostly depend on the certain application domain and are designed so that they address certain needs, e.g., in the solution for the rehabilitation domain [57], to perform a particular interaction, one must physically move (e.g., sit and stand). Additionally, in some solutions from the visual disability category an additional output modality occurs–a haptic vibration, which usually activates to indicate that a user is progressing in the application, e.g., when finding an object or the right direction in navigational applications [62].

### 4.4. RQ4: What Accessibility Features Have Been Implemented in Handheld AR Solutions for People with Disabilities?

Given that AR solutions included in this review are developed for people with disabilities, the solutions are accessible at least to the target group of users. Thus, we extracted accessibility features that can be considered good practices for addressing AR accessibility challenges when developing handheld AR solutions for everyone. Accessibility features shown in Table 4 are grouped according to the existing WCAG accessibility principles (*Perceivable*, *Operable*, *Understandable*, and *Robust*) as they are highly relevant to both web and non-web mobile content and applications [20]. In the second column of the table, to which category of requirements the accessibility feature refers is indicated, e.g., *multi-modality* is one of the requirements emphasized in the XAUR document as one of the ways for fulfilling diverse user needs related to accessibility in XR [23]. Additionally, for each publication, which age group and disability type the solution was developed for or evaluated with is indicated.

The accessibility features in the *Perceivable* group address the user needs related to making AR content and UI elements presentable to users in ways they can perceive them. Providing more than one input or output modality allows users to perceive the content in the form they need, e.g., by providing text alternatives for any non-text content. In addition, if there is an option to adjust the content by changing the color, contrast, font type and size, or by providing an alternative screen display, users will have a more accessible representation of all the information they need to make the best use of the application. In the *Operable* group, there are accessibility features related to UI elements and navigation through screens that allow the user to perform the required interaction. For example, in some solutions, an audio interface works alongside the visual interface, enabling hands-free interaction if speech recognition is implemented. This group also includes UI elements with features that are necessary for some users to interact more easily, such as larger buttons or buttons with large interactive touch zones, alternative elements in case it is difficult to use the default ones, or additional features such as the *Freeze* feature. The accessibility features in the *Understandable* group refer to making information and the operation of UI comprehensible to different user groups. To make content more understandable, some might request more simplified text, while others might appreciate easily recognizable icons for buttons or animations to explain tasks. A general design guideline for the UI would be that it is intuitive and consistent, and this is no exception in AR solutions. The accessibility features that belong to the *Robust* group are any that meet the requirement that the content can be interpreted by a variety of user agents, including assistive technologies, which is the case for all solutions supporting screen readers, so they are not listed in the table. Publications that are not listed in the table and from which accessibility features have not been extracted mostly refer to AR solutions that use the Aurasma application, which is an authoring tool (i.e., used to create custom markers and “auras” as AR content) and it is not possible to add features other than scanning the marker, or AR navigation applications (i.e., those that use the navigator heads-up display).

### 4.5. RQ5: What Research Methods Are Applied in the Design of Handheld AR Solutions for People with Disabilities?

In this section, we present a summary of methods used in the research, design, or evaluation of handheld AR solutions for people with disabilities. In addition to presenting methods and techniques used in the research and design of evaluated solutions, we also mention what data were collected to confirm the usability of these solutions (for those papers that conducted an evaluation). Table 5 shows the research methods and the classification of studies by disability. The studies we classified are grouped according to the following research methods: *Qualitative*, *Quantitative*, and *Mixed*. In the table, the column entitled *Data collection* refers to the techniques in the phases of requirement collection or solution evaluation to collect data. About 90.6% of the systematic literature review publications indicated which methods and/or techniques they used in the research design or evaluation of the AR solution. The definitions for qualitative and quantitative methods were taken from [70]. The percentage of studies that used qualitative methods is 27.6%, while 65.5% used a mixed method approach. Only 6.9% used quantitative methods, as shown in the next table (Table 5).

In terms of metrics collected during application testing, researchers in [51] measured performance metrics such as reading speed, as well as preferences in fonts, size, text-to-background contrast ratio, line height, etc. The usability analysis conducted in [54] collected data such as task completion, number of errors, the time required to complete the task, user rating of the experience, and amount of instruction required. The results of studies [54,61] show that previous experience with the technology is as important as instructions or tutorials for those who do not have experience. To avoid system errors, various challenges that users may face (such as lighting conditions affecting marker accuracy [55] or latency and processing time in collaborative AR games [69]) should be considered to minimize hazards and the negative consequences of accidental or unintended actions. ARCore technology can recover from surface-tracking errors by restoring the previous positions of virtual objects after the environment is re-detected [69]. Participants in a user study described in [39] used prototypes of non-visual alternatives to common AR tasks over a series of five tasks. Apart from participants’ responses to the question, only time per task or use of the application was measured during the procedure. Various contextual parameters such as the size and complexity of the physical environment and the user’s level of familiarity with the space emerged as another factor affecting the usability. In different cases, different interaction methods may be easier to use, so it would be useful to investigate how AR interactions can be automatically identified and adapted to be accessible [39]. In a case study described in [68], initial usability and acceptability tests were conducted. Quantitative metrics collected included execution time, number of errors or percentage, and task completion, while qualitative metrics were related to perceived ease of use and comprehensibility of information. The results showed the great potential of AR as an assistive technology in manufacturing.

Future evaluations in similar research could be based on artificial intelligence, as is the case in [47], which describes a practical implementation of the AR interface for teaching students with special needs. The application described in [47] uses a neural network to assess learners’ practical knowledge and is expected to be further enhanced using machine learning to generate specific tips to help students with disabilities learn more effectively. These novel uses of technologies open up the possibility of creating the educational environments necessary to address the individual needs and requirements of each student by generating recommendations and automatically adapting the learning environment.

### 4.6. Implications of Accessibility Good Practices Derived from the Review

The accessibility features described in Section 4.4 are related to user requirements identified in AR applications in terms of multi-modality, content (virtual and UI), assistive technology support, and instructions. However, some other features and design guidelines can be considered good practices and recommendations in the domain of accessible application development and design in general. In this section, we highlight some of them by describing the features and results of the AR solutions investigated. We also mention if any specific problems or challenges were encountered during the study. The examples are organized by disability category.

#### 4.6.1. Cognitive, Learning, and Neurological Disabilities

Authors in [40] emphasize a device-specific feature (called *Guided Access*) for iOS devices that allows disabling the *Home* button and touch functionality of some parts of the screen. This can be helpful to pupils with autism, attention disorder, and mental disabilities to better concentrate on the task.

In the study described in [41], researchers have used the combination of a user-centered and collaborative design process as a methodological approach for developing mobile AR applications. They relied on universal design for learning as an inclusive educational approach, so the application incorporates principles for achieving a learning environment for all by including multiple means of presentations, such as icons with alternative text, images, texts, and videos with subtitles, as well as multiple means of expression and engagement. Similarly, researchers in [50] collected requirements for the development of an AR framework with gamification to assist the learning process of children with intellectual disabilities through interviews with different experts. Requirements gathered in this study are related to learning content, interaction design, and technology used, and can be a good example of achieving universal access to knowledge.

Analysis of the evaluation results for the next AR solutions derived some useful findings that can be taken into consideration in the development of new solutions. For example, evaluation results in [42] showed that visual prompts in navigation applications help students with intellectual disabilities, i.e., students benefited from real-time visual prompts, while the results of students in [43] showed that providing cues affected their performance improvement. Similarly, color matching (a border around the recognized object and a corresponding button), as well as audio feedback, showed important for easier use of application for autistic children in [45]. An application proposed in [46] showed the ability of simple novel technologies to be used as effective rehabilitation tools for adults with autism. For such solutions, it is important to keep the features and interaction model simple and easy to use and to include achievement rewards to increase motivation and performance monitoring. When considering the design of the application in [47], the researchers took into account that children with autism are very technocritical and love gadgets more than communicating with teachers, so they chose a robot as an avatar that guides the child through the application.

Some relevant findings regarding design concepts for learning environments can be found in [48], as the study results showed that the gamified elements implemented in the application highly increased the motivation of learners with special needs. Additionally, the graphical UI was perceived as child-friendly and aesthetically pleasing. One of the findings was also that mentally disabled children of the same age differ greatly in attention span, which is one more implication that an individual approach is important.

One of the findings in [49] for enhancing the user experience in an application for students with intellectual disabilities is to add more interactive features, although an overall improvement in automatic teller machine skills was demonstrated in the experiments. This application is based on the HP Reveal authoring tool, which does not offer many possibilities for interaction with the augmentations, which indicates that different AR technologies should be considered in further research for this target group. In the study described in [52], an AR application served as a means for delivering a video-based intervention to individuals with Down Syndrome. Participants said that the iPad and the HP Reveal were easy to use. Although the previous experience with iPad usage helped, two out of three participants reported difficulty with double tapping the videos to make them full screen. An additional button with full-screen functionality would certainly be helpful.

In the study described in [53], there were also some challenges in operating the device, i.e., holding the device too close to the marker or placing the hand over the camera, which is then handled with assistance or verbal explanation. To allow the student to work independently, it would be useful to show them instructions on what to do next. Since people with Down Syndrome are visual types, it would be beneficial for their attention to avoid large text and replace it with illustrative images or audiovisual content, as described in [56].

In an empirical study described in [38], older participants showed overall satisfaction with the CogARC system which was developed based on the UD principles and the suggestions for design and usability for older players. However, some of the key findings are that age-related changes in cognition should be considered when implementing AR for older users and that an iterative design approach is necessary according to their experience. In addition, the interaction technique, which affects the perception, cognition, and emotional state of the user, proved to be an important component of the developed system. Some of the AR-specific challenges which are addressed in [38] are the marker occlusion problem (the user’s hand hindered tracking, which was solved by developing a multi-marker setup), the lagging issue (delay between the actual movement of the player’s hand and its display on the screen via the AR camera capture), the limited gaming space defined by the small screen size (10”), and the player’s loss of depth perception while looking through the tablet screen (which was solved by an initial session of familiarization with the system’s interaction technique and game content).

#### 4.6.2. Physical Disabilities

When designing interaction techniques so that people in wheelchairs can use the application, it is necessary to consider the position of markers if the application is marker-based (in this case, the markers should be at the height of a user [58]). Additionally, the application should not require a lot of movement, as they are constrained. Additionally, if a user has low hand mobility, i.e., hand tremor, the recommendation is to use touchscreens with large fonts and interfaces [59]. Additionally, in [59], the researchers hypothesize that a smartphone is a better choice than a tablet, as they found some grasping and managing issues with the tablet for users in wheelchairs and with regular hand movements. Other important aspects of interface design are mentioned in [69]. One is related to mobile device rotation and display adjustment depending on device orientation, and the other is related to enhancing the user experience for children through haptic feedback used to reinforce the system’s response to user interactions.

#### 4.6.3. Visual Disabilities

Research on image-enhancement applications for people with low vision has investigated various image transformations, including contrast enhancement, color transformation, intensity-edge enhancement, and magnification and image stabilization, as mentioned in [63]. Assistive systems for blind mobility usually provide non-visual (auditory) feedback [61], but when thinking of solutions that are accessible to all, all requirements must be accommodated in one solution. As mainstream AR technologies continue to advance, accessible spatial information will be available faster, in more contexts, and at a lower cost than ever before. This is promising in the context of increasing the number of applications such as *overTHERE*, described in [63]. A more detailed description of the mobile AR applications’ accessibility for the visually impaired can be found in [39], where the authors describe a way to make accessibility services aware of virtual content and allow developers to assign metadata to it. Additionally, the study opened many challenges and areas for future research to make AR fully accessible.

#### 4.6.4. Auditory Disabilities

An application described in [64] is intended for understanding and individual practicing driving scenarios of young people with hearing disabilities. Although it does not have accessibility options per se, possible alternative features can be considered to make the application more accessible. For example, the arrangement of UI buttons for car manipulation, i.e., grouping all buttons on one side or arranging the buttons for a certain type of manipulation at different sides of the screen, can facilitate interaction depending on how the user holds the device or in which orientation.

#### 4.6.5. Multiple Disabilities

One of the rare AR solutions that has implemented accessibility options is the one described in [66]. Accessibility options are displayed at the first launch but can be adjusted later in the settings, and the choice remains persistent between application usages. It is also important to offer different difficulty levels or AR assistance to children with different disabilities and special needs, as is the case with the AR application in [67]. This builds confidence and increases the possibility of completing the game. Given that different disabilities or even the same ones require different user needs, the personalized use of an application is preferable. A great example of an adaptive UI for the elderly, who often face various difficulties as they age, is described in [68]. They configure the UI features and select the appropriate content depending on the user profile and contextual information. Some interesting features implemented by the games described in [69] are: showing and hiding the main menu (which can be helpful to see the whole screen in AR), restarting the game, scaling and rotating virtual content to see it from a more comfortable point of view, and adjusting the size and position of the scaling and rotation sliders when rotating the mobile device, which can be considered good practice for mobile devices and UI.

All of the above findings are a good start for further research in the field of inclusive AR applications, as there are no concrete guidelines on how to apply them to AR content or new interactions required in AR applications. As this review has shown, there are attempts to make content and interactions more accessible in applications developed for specific audiences. However, there is a lack of concrete steps for developers and designers to develop AR applications accessible to everyone.

## 5. Discussion and Future Work

This systematic literature review is conducted with the goal of answering five research questions. At the beginning of the discussion, the results of a bibliometric analysis are commented on. It should be noted that no publications with relevant information for the field of accessibility in mobile augmented reality (MAR) solutions were found before 2015. The average number of 4.5 publications per year since then indicates that awareness should be raised for the development of accessible MAR solutions for all. Publications in this review were considered based on their relevance to the field of accessibility, i.e., if they included a description of the solution used in the study with interaction techniques and/or features relevant to improving the accessibility of the solutions. Since these solutions were developed exclusively for people with a specific type of disability (or multiple disabilities), interaction techniques and additional features are examined in more detail, as they can be considered important if users were satisfied and rated them as usable/accessible.

The most popular type of mobile AR solution for people with disabilities in the last decade is marker-based solutions, which use markers (different types of triggers) for establishing correspondence between the physical and virtual worlds. As with most solutions used in education, the majority of triggers for marker-based solutions are located in a textbook or electronic book, on flashcards or learning objects, or in the form of images associated with the learning material or other visual markers. The next-most popular type is location-based solutions used to support navigation or wayfinding. The primary trigger signal here is GPS, and this is also true for hybrid solutions that use inertial tracking triggers in combination. Very few solutions are based on markerless tracking, although this is a more flexible form of AR than the marker-based method. Considering that this technology is at present developed to improve the experience of AR and that it allows object occlusion, better immersion, and novel interactions, this tracking type should be better explored in terms of use by people with disabilities.

Considering that cognitive, learning, and neurological (CLN) disabilities can affect the ability to move, hear, speak, and understand information, this category includes the most diverse group of users, and most solutions are found for this category of disabilities. Solutions for visual impairments are found only in the navigation and assistive tool categories, meaning they are underrepresented in other application areas. In general, the technologies used for game development still do not provide adequate support for assistive technologies such as screen readers, and accessibility-aware developers must rely on their efforts to do something about it. Another complicating circumstance is that there are no guidelines or standards for how AR content can be made visible to assistive technologies. Therefore, additional efforts should be made to explore this area so that people with visual impairments can use applications in a variety of settings. The category for which the fewest solutions have been developed, and which therefore has the greatest potential for further research, is auditory disabilities. The reason for this could be that people who are deaf or hard of hearing can perceive information primarily visually, and many applications contain instructions only in text form. Content in text form is not useful especially for people who are deaf from birth, as they often cannot read. In this case, a highly simplified text, illustrations, or sign language can be helpful for them, which is difficult to implement in such a solution, especially considering that each country has its sign language or the content changes dynamically.

Most augmentations in analyzed solutions are visual, and range from on-screen information in the form of 2D graphics, text, and icons that appear on the screen (mostly in navigation, instruction, or information applications) to various images, animations, videos, and 3D objects. While 3D models representing different objects from the real or virtual world are expected to provide more opportunities for interaction, other types should not be neglected. All augmentation types should take into account different aspects of accessibility, such as applying different accessibility options depending on the user’s needs (changing contrast, font, highlighting edges, etc.). This is especially important for educational applications where different AR content is used as a means of learning school subjects, or for navigation applications where it is important to find the right path in an unfamiliar environment.

Given that almost all of the handheld AR solutions for people with disabilities studied use an AR information-browser type of interface, further research efforts can be made to investigate the potential of other types of interfaces for people with disabilities, whether it is a primary interface or an alternative interface for users with disabilities. When looking at the tasks identified in each solution, it is interesting to note that for the most numerous solution category by disability (CLN), no tasks were found for scanning the environment, transforming virtual content in the form of scaling and color change, and the task of selection. Similarly, the solutions for people with physical disabilities did not include any tasks for scanning the location and placing a virtual object. Additionally, the solutions for people with visual impairments did not require the user to scan the markers or the object or transform the object in any way. All of this suggests that further efforts are needed to include people with different disabilities in the development of solutions so that they comply with the principles of universal design and allow equal use of all solutions without restrictions on certain tasks that “they cannot do” or can do if there are alternative ways to do them.

In AR applications, different types of interactions may be required of the user to perform certain AR tasks, such as scanning a marker, object, or environment; placing, moving, or rotating an object; or triggering certain effects. Depending on the type of application and the disability for which a solution is developed, different interaction techniques are used for specific AR tasks. Since tasks involving scanning are the most common, it is worth looking at the interaction methods required for this interaction task. Most scanning tasks involve the *Pointing to* interaction technique, which requires the user to hold the device with one or both hands and point the device’s camera, usually located on the back of the device, at the target if a marker-based technique is used, or at the environment to establish physical–virtual correspondence if a markerless technique is used. Depending on the application, the device can be used in portrait or landscape orientation. Considering that some people have motoric difficulties, whether they have limited movement or can only use one hand, it would be beneficial for their inclusion and sense of equal participation regarding their limitations to find a way to perform this type of AR task. It is challenging for people with visual difficulties to determine their exact location when they do not know what is in their immediate and wider surroundings. One of the guidelines for their inclusion would be to provide them with audio descriptions of their surroundings and guide them on what to do. For people with cognitive or learning disabilities, one guideline would be to include simple animations that show them exactly what to do to establish a physical and virtual correspondence so they can continue to use the application. Just these few examples show that it would be beneficial to have alternative ways to use the application that meet different user needs. This can be enabled by providing appropriate accessibility features in the application settings or by including them in the design of the application during implementation.

User needs may depend not only on the type of disability but also on the age of the user. While it is important for most AR solutions for children to provide multiple means of representation, especially for solutions intended for learning, for solutions intended for adults it is important to ensure support for assistive technologies such as screen readers or to provide alternative input methods such as voice commands. While older users may appreciate large game buttons, it would be beneficial for all age groups to provide accessibility options to change the appearance of content from UI and AR in terms of color (contrast), font type, and size, as well as to provide clear instructions. Many different requirements need to be considered to meet different user needs. This is a very complex task, as these requirements can be contradictory, even if they are found in the same age or disability category. This is also mentioned in [50] for people with intellectual disabilities, as they form a heterogeneous group and their cognitive and adaptive limitations vary from person to person. Individual needs (physical and cognitive impairments) should be taken into account when designing systems for stroke rehabilitation, which is highlighted in [57] as one of the criteria for designing systems for stroke survivors. In addition, the research described in [51] has shown that there is no universal solution in terms of font types, sizes, spacing, and contrast for people with dyslexia. Therefore, personalization at the individual level is very important to improve both accessibility and user experience.

In [68], a case study is described that uses personalization of the user interface according to the user’s skills, tasks, age, and cognitive and physical abilities. They use a set of knowledge-based rules to configure the components of UI (e.g., the type of content to display, the amount of content displayed, and the characteristics of the interface in terms of font, text, icons, color). The importance of adaptability to user needs and interests in a given context in real time was also recognized by researchers in [54], who described an adaptive AR system and proposed their adaptation model. While various surveys can provide general information about the game experience and the perceived usability of the system, they cannot directly help identify technical, gameplay, and usability issues [38]. In addition to conducting user interviews to identify elements that impact playability, tracking various objective metrics in a game could identify specific usability issues as well as different user preferences if subjective evaluation is integrated into an app. An adaptive system with AR applications that relies on user behavior analysis could be used to customize and set up an AR application to meet user needs and preferences. For example, if a game with default interaction techniques presents certain interaction problems for a user, the system could offer the user an alternative way to interact or view content the next time they use the application. This is something we would like to explore further in the future in the field of accessibility and the consideration of user capabilities and contextual information.

The motivation for the proposed future work is described in this section. The authors of the review article [71], which provides a comprehensive overview of AR user studies conducted between 2005 and 2014, encourage researchers to continue the approach of qualitative and quantitative evaluation to assess the usability of AR applications. They also emphasize the importance of capturing as many different performance metrics from the user study as possible to fully understand how a user interacts with the system (i.e., not just task-completion time). Furthermore, as the use of serious games and game-based learning increases today, game-learning analytics provide an interesting way to track user behavior and evaluate performance to make suggestions for adapting the content and interaction type to facilitate game use. Existing game-learning analytics platforms allow for the tracking of various metrics and user data so that they can be used for different purposes. As stated in [62], time and resources are required to analyze and study user behavior and needs. A combination of AR serious games for learning, game-learning analytics, best approaches for evaluating the usability of AR applications, and the findings from this review provides a good basis for further research aimed at customizing AR handheld solutions to the user in terms of accessibility, which is important for people with disabilities in general, the elderly, and especially children who cannot usually customize the solution themselves.

## 6. Conclusions

Accessibility is an important aspect of achieving social and digital inclusion. While mobile augmented reality has already entered our lives, not enough attention has been paid from the beginning to how AR solutions can be made accessible. This paper aims to provide a review of handheld AR solutions developed for people with disabilities between 2010 and 2022 that are relevant to the field of accessibility. The results of this review help us understand what interaction tasks and techniques in handheld AR solutions are required from users with different disabilities to find research gaps in terms of accessibility that need to be addressed in future work. The accessibility features of each solution are explored and extracted together with other accessibility good practices. Certain accessibility features can be beneficial to all users, e.g., voice commands that enable users with different disabilities to manipulate the augmentations will also allow hands-free operation for all users.

Considering the very heterogeneous needs of users with permanent or temporary disabilities, it is a very complex task to develop a solution that fulfils all their, usually conflicting, needs. Besides having in mind the accessible design of the solution, for AR solutions on mobile devices, one must think about accessible interaction techniques, which is not a clear-cut decision, since different users may prefer different interaction types. To know which user group prefers which interactions in the context of accessibility, tracking objective and subjective measures while a user uses the application imposes itself as a possible solution. Besides the input method required to perform a certain task in the AR solution, different contextual factors can affect the usability and accessibility of an AR solution, such as different environmental factors (e.g., different lighting conditions or types of surfaces, noisy places) as well as the size of the device (e.g., smartphone, tablet) or internet access. All of these must be considered when defining accessible interaction techniques and content display for a certain user.

By employing some simple principles that apply to website accessibility, developers can make AR solutions more accessible to people with disabilities. Making AR solutions accessible to as many users as possible requires more thought about accessible interaction options. By allowing alternative options for interacting and viewing content, we are getting closer to a solution that is designed around the principle of universal design, which is the goal for all solutions that people use every day. The results of this literature review will aim at formulating guidelines that will enable developers and designers to seamlessly achieve the ultimate goal we should all strive for, which is the social inclusion of children and people with disabilities, as well as the elderly, using emerging technologies that have already proven useful in a variety of settings.

## Figures and Tables

**Table 1:** AR tracking technology categorization based on the sensor/technique used for tracking in AR mobile tracking systems.

AR Tracking Technology Category	Tracking Technology Type by Sensor/Technique		Tracking Target (Trigger) Examples
Vision-based tracking (Optical tracking)	Camera	Marker-based tracking	Fiducials (visual markers), tag-based (barcode)
		Markerless tracking	Natural features (model-based, model-free)
Location-based tracking	GPS		GPS signal
Inertial tracking	Gyroscope		Angular velocity
	Magnetometer (Compass)		Orientation relative to the Earth’s magnetic field
	Accelerometer		Acceleration
Hybrid	Combination		GPS signal and inertial tracking triggers

**Table 2:** Identified task types in every publication categorized by task category and disability type.

		AR Task Category										
		Establishing Physical/Virtual Correspondence				Placing Virtual Content	Transforming Virtual Content				Activating Virtual Content	
Disability	Publication	Scan Marker	Scan Location	Scan Object	Scan Environment	Place Object	Move	Rotate	Scale	Color	Select	Trigger Effect
Cognitive, learning and neurological	[40]	✓										
	[41]	✓										
	[42]		✓									
	[43]		✓									
	[44]	✓										
	[45]			✓								✓
	[46]			✓			✓					
	[47]	✓										
	[48]	✓										
	[49]	✓										
	[50]	✓										
	[51]	✓										
	[52]	✓										✓
	[53]	✓										
	[54]		✓									
	[38]	✓				✓	✓	✓				
	[55]	✓										✓
	[56]		✓	✓								✓
Physical	[57]			✓	✓		✓					✓
	[58]	✓										
	[59]	✓									✓	
	[60]	✓						✓	✓			✓
Visual	[61]				✓							
	[62]				✓						✓	✓
	[63]		✓								✓	
	[39]				✓	✓					✓	
Auditory	[64]	✓				✓	✓					
	[65]	✓										
Multiple	[66]	✓						✓	✓	✓		
	[67]	✓										
	[68]	✓										
	[69]				✓	✓		✓	✓		✓	
%	100	62.5	15.6	12.5	15.6	12.5	12.5	12.5	9.4	3.1	15.6	21.9

**Table 3:** Interaction techniques and description of input methods for performing the tasks by task type and disability category.

Task Type	Disability Category	Interaction Task Technique	Input Method Description	Publications
Scan marker	CLN	Pointing to scan	Hold and move the device to point at the marker/image/text to show augmented information	[40,41,44,47,48,49,51,52,53,55,58,59,60,64,65,66,67,68]
	Physical			
	Auditory			
	Multiple			
	CLN	Set marker	Bring marker(s) in front of the camera	[50]
		Set tangible	Move AR cube(s) under the device’s camera	[38]
Scan location	CLN	Pointing to location	Move the device camera around to find the direction of the target location (camera towards horizon or eye level)	[42,43,54,56]
	Visual	Point in POI direction	Hold and move the device to point in a direction of a place of interest to hear additional information	[63]
Scan object	CLN	Point camera around	Move the device camera around to recognize object	[45,46,56]
	Physical	Point to target	Move the device to aim the target	[57]
Scan environment	Physical	Point camera around	Move the device with camera to establish the environment	[57,69]
	Multiple			
	Visual	Point camera around	Hold the device to recognize the environment and show or play additional information	[39,61,62]
Place object	CLN	Place tangible	Move AR cube under the device’s camera	[38]
	Visual	Camera-based placement	Virtual objects follow the phone’s position; user confirms location by clicking on a button	[39]
		Guided placement	Objects are automatically placed and rotated based on the user’s selection from different options (user is instructed)	[39]
	Auditory	Set scenario (place marker(s))	User models game scenario by placing markers in real space and configuring position of the game object	[64]
	Multiple	Detect surface	Where camera ray cast and surface collide, there the virtual object will be placed	[69]
Move/Translate	CLN	Drag and drop	Hold the device and perform drag and drop gesture on screen	[46]
		Move tangible	Move AR cubes next to each other physically	[38]
	Physical	Move physically	Stand to collect and sit to throw virtual object while aiming	[57]
	Auditory	Click	Manipulation of game object by clicking on UI buttons on screen (left, front, back, right)	[64]
Rotate	CLN	Rotate tangible	Rotate AR cube in space	[38]
	Physical	Voice command	Rotate a 3D model by holding a voice recording icon on screen and saying “Rotate”	[60]
		Horizontal slide gesture	Perform horizontal slide gesture on screen to rotate	[60]
	Multiple	Click	User changes the rotation speed of the object by clicking on UI buttons	[66]
		Slider	User changes the rotation (speed) of the object by moving the handle on a slider	[66,69]
Scale	Physical	Voice command	Scale an image or video by holding a voice recording icon on screen and saying “Enlarge” or “Shrink”	[60]
		Pinch to zoom	Perform pinch to zoom gesture on screen to scale an image or video	[60]
	Multiple	Click	User changes the size of the object by clicking on UI buttons	[66]
		Slider	User changes the size of the object by moving the handle on a slider	[66,69]
Color	Multiple	Click	User changes the color of the object by clicking on UI buttons representing different color	[66]
Select	Physical	Click	Hold the device and click on a specific product/target	[59]
	Visual	Tap	Hold the device and tap on the visualized location point on screen or item from the list	[39,62]
		Voice command	Select a destination by saying an appropriate command	[62]
		Click	Hold the device and click on a location from the list to pinpoint it or add virtual sign	[63]
		Camera-based search	When an object is in view of the camera, information about it is spoken with haptic vibration as well	[39]
	Multiple	Tap	Tap on the 3D object on screen to select it	[69]
Trigger effect	CLN	Click	Hold the device and click on UI button to play sound/video	[45,56]
		Double tap	Double tap to enter fullscreen	[52]
		Tap	Trigger an animation or sound by tapping a virtual object on screen	[55]
	Physical	Move physically	Sit to perform action with a virtual object	[57]
		Voice command	Start or stop video by holding a voice recording icon on screen and saying “Start” or “Stop”	[60]
		Tap	Tap on video to start or stop it	[60]
	Visual	Voice command	List the destinations around the user by saying the appropriate command	[62]
			Activate the navigation instructions by saying the appropriate command	[62]
		Correct direction	User triggers vibration motor when facing the correct direction when following navigation instructions	[62]

**Table 4:** Accessibility features implemented in AR solutions included in the review grouped by WCAG principles.

Principle	Requirement Category	Accessibility Feature in AR Solution	Participant’s Age (Years)	Disability Category
Perceivable	Multi-modality	Visual and audio feedback (for learning) [48]	5–10	CLN
		Alternative text display (*Reader Mode*) with option to read the text aloud [51]	12–14	CLN
		Spoken (voice) instructions—to improve understanding and interaction (especially for those who cannot read) [50]	Children	CLN
		Visual and audio support for navigation [61]	Adults	Visual
		Visual, audio and haptic feedback [62]	Adults	Visual
		Image, video, and sound representations of content [65]	7–12	Auditory
		Multiple means of representation (3D model in AR with textual and sound descriptions) [66]	Primary school	Multiple
	Assistive technology	Support for text-to-speech function [60]	Students	Physical
		Screen reader support (text alternatives) [61]	Adults	Visual
		Screen-reader support (VoiceOver)—3D virtual objects transformed into 2D voice over targets [39]	Adults	Visual
	Content	Changing text to sans-serif font, higher color contrast ratio between background and the text, and increased letter spacing [51]	12–14	CLN
		Alternative text display (*Reader Mode*) with clear and bigger text [51]	12–14	CLN
		Game objects used as augmentations have clear elements highlighted through contrast and color settings [38]	Elderly	CLN
		Customization of font style, UI theme color (with different colors of text, background, and buttons for better contrast ratio) [66]	Primary school	Multiple
		High contrast option for displayed AR models [66]	Primary school	Multiple
	Instructions	Player guided by text and icons (game’s auditory feedback is limited) [38]	Elderly	CLN
Operable	Multi-modality	Support for touch and voice input methods [58]	Adults	Physical
		Support for speech recognition (voice commands allow hands-free operation) [60]	Students	Physical
		Screen reader support (navigation with TalkBack) and voice sign-in [61]	Adults	Visual
		Visual interface and auditive input (voice recognition) [62]	Adults	Visual
	User interface	(Help) button on the screen with large interactive touch zones [57]	Avg. 70	Physical
		Large play buttons [56]	Adults	CLN
		Alternative interaction elements (buttons instead of sliders) [66]	Primary school	Multiple
		Additional interaction features (rotation and scaling) [69]	Children	Mobility constraints
	Content	Freeze feature—captures a stable view of the AR content and the physical world it is overlayed onto [39]	Adults	Visual
Understandable	Content	Simplified text description (plain language) [53]	19–25	CLN
	Instructions	On-screen instructions to help users understand what to do (e.g., perform specific interaction/task) [46]	25–50	CLN
		Verbal notifications while performing an activity [39]	Adults	Visual
		Pop-up window informing user about the action (especially for color-blind people) [66]	Primary school	Multiple
	User interface	Clear and simple menus, large-sized icons, text, and buttons [38]	Elderly	CLN
		Buttons with conventional and recognizable icons [56]	Adults	CLN
		Readable and consistent interface with familiar experience [57]	Avg. 70	Physical
		Simple and intuitive interface design, small scope of items for reading [61]	Adults	Visual

**Table 5:** Research methods and data-collection techniques used in AR solutions by disability.

Research Method	Study Classification	Data Collection	CLN	Physical	Visual	Auditory	Multiple
Qualitative	Case study User study	Video recordings, photography, text notes, asking questions	[40]		[39]		
	Cross-sectional study	Questionnaire, direct observation (recorded), IMMS ^1^ instrument	[41]				
	Single-subject case design	Event recording procedures; not specified	[42,43,44]				
	Field study	Screen recordings, worksheets, interview	[48]				
	Exploratory study	Interview, observation, questionnaire, focus groups	[56]				
Mixed	Multiple baseline or probe design	Data sheets, video recording, observation, survey	[49,52,53]				
	Usability analysis	Objective measures, questionnaire, neural network, survey	[47,51,54,55]		[61]		[66,68,69]
	Empirical study	Questionnaires, interview, observation; thinking aloud, photographs, videos; TAM ^2^	[38,57]	[59,60]			
	Comparative user study	Questionnaire, interview, user observation				[65]	
	Pilot study	Opinion, questionnaire, survey	[45]		[61]	[64]	
Quantitative	Experimental study	Questionnaire, survey	[46]				[67]

^1^ Instructional Materials Motivation Survey (IMMS); ^2^ Testing Acceptance Model (TAM).

## Data Availability

Not applicable.
